# Health Information Orientation Profiles and Their Association with Knowledge of Antibiotic Use in a Population with Good Internet Access: A Cross-Sectional Study

**DOI:** 10.3390/antibiotics11060769

**Published:** 2022-06-04

**Authors:** Huiling Guo, Huai Yang Lim, Angela Chow

**Affiliations:** 1 Department of Preventive and Population Medicine, Office of Clinical Epidemiology, Analytics, and Knowledge, Tan Tock Seng Hospital, 11 Jalan Tan Tock Seng, Singapore 308433, Singapore; huiling_guo@ttsh.com.sg (H.G.); lim_huai_yang@defence.gov.sg (H.Y.L.); 2 Saw Swee Hock School of Public Health, National University of Singapore, 12 Science Drive 2, Singapore 117459, Singapore; 3 Head Quarters, Singapore Armed Forces Medical Corps, 701 Transit Road, Singapore 778910, Singapore; 4 Lee Kong Chian School of Medicine, Nanyang Technological University, 11 Mandalay Road, Singapore 308232, Singapore

**Keywords:** antibiotic stewardship, antimicrobial resistance, general population, health information orientation, knowledge of antibiotic use

## Abstract

Background: Poor knowledge of antibiotic use drives poor antibiotic practices, but little is known about the influence of health information orientation (HIO) on knowledge of antibiotic use in the general public. Methods: We conducted a nationally-representative population-wide cross-sectional study (November 2020–January 2021), on a proportionately stratified random sample of 2004 Singapore residents aged ≥21 years. Multivariable logistic regression analysis was performed to assess the association between HIO and knowledge of antibiotic use. Results: Forty percent of respondents had low-levels of HIO (LL-HIO); they tended to be younger, not currently married, and did not have family/friends working in the healthcare sector. Respondents with LL-HIO (aOR 1.82, 95% CI 1.32–2.51, *p* < 0.001) were 82% more likely to have poor knowledge of antibiotic use. In particular, older adults aged ≥50 years with LL-HIO (aOR 1.81, 95% CI [1.32–2.51], *p* < 0.001) were much more likely to have poor knowledge than their HL-HIO counterparts. They were also less likely to use the Internet to seek health information and had poor eHealth efficacy. Conclusion: LL-HIO is independently associated with poor knowledge of antibiotic use. Educational strategies on antibiotic use should disseminate a consistent message through both online and offline platforms, involving traditional and non-traditional healthcare and non-healthcare influencers.

## 1. Introduction

The discovery of penicillin by Sir Alexander Fleming in 1928 heralded the era of antibiotics, saving countless lives by treating bacterial infections in humans. However, the often poorly regulated and widespread use of antibiotics, coupled with the lack of discovery of new antibiotic classes since the 1980s [1], have led to the imminence of a post-antibiotic era where increased drug resistance renders antibiotic treatment ineffective [2,3]. Lack of public awareness and knowledge of antimicrobial resistance (AMR) and poor knowledge of appropriate antibiotic use are major drivers of inappropriate antibiotic practices [4,5,6,7,8], prompting the World Health Organization (WHO) to list the improvement of awareness and understanding of AMR as one of the five key strategies in its 2015 Global Action Plan on Antimicrobial Resistance [9].

Despite various initiatives to raise public awareness, systematic reviews found that population-wide traditional mass-media AMR campaigns to educate the general public had not resulted in significant improvements in appropriate antibiotic use [10,11,12,13]. A more nuanced approach utilising a combination of strategies that target different sub-populations is evidently necessary to address knowledge and practice gaps, and improve antibiotic stewardship in the community. This is further supported by a previous study reporting the modifying effects of age on the association between poor knowledge of antibiotic use and AMR, respectively, and poor antibiotic practices, highlighting the need to tailor educational interventions by age [14].

The presence of online and social media platforms in this digital era has greatly shaped the health information seeking behaviours (HISBs) of the general public, as these platforms provide more convenient and time-efficient options to seek out answers. Most online health information consumers reported success in obtaining answers to their health-related questions and more than one-third (40%) believed that online sources of information are credible [15]. As such, online and social media platforms could serve as alternative health information dissemination modalities to the more traditional forms of mass campaigns, posters, brochures and advertisements through mainstream media.

Health information orientation (HIO) refers to an individual’s intrinsic interest in seeking out relevant health information for the purposes of making informed decisions regarding one’s health [16]. A high level of HIO (HL-HIO) indicates a desire to increase self-efficacy through the acquisition of relevant knowledge and information. In contrast, individuals with a low level of HIO (LL-HIO) demonstrate a lower proclivity to seek out health information and prefer to receive health information passively. While associations between HIO, HISB and health behaviours have been described [17,18,19], little is known about the influence of HIO on health knowledge, in particular, knowledge of antibiotic use.

Therefore, in this study, we sought to use a nationally representative population-wide cross-sectional survey to (1) explore the relationship between HIO and knowledge of antibiotic use, and (2) understand the online HISB of sub-populations with different levels of HIO, to identify educational delivery platforms to improve the knowledge of antibiotic use in the general public.

## 2. Materials and Methods

### 2.1. Study Design and Sampling Frame

A nationally-representative population-wide cross-sectional survey was conducted between November 2020 and January 2021, on a proportionately stratified random sample of Singapore citizens and permanent residents aged ≥21 years. Households were randomly selected from addresses with at least one Singapore resident, after stratifying by their predominant race, followed by broad dwelling type. Malays and Indians were oversampled to ensure representation of these minority groups in the final dataset. The household member with the most recent birthday was invited to take part in the study.

### 2.2. Survey Data Collection

The survey package consisted of a hardcopy survey booklet, with instructions on how to complete the survey and also an optional web link for participants who preferred to complete the survey online. The survey was predominantly self-administered and disseminated via a drop-off/pick-up method. However, interviewers were also trained prior to data collection to administer the survey in a standardised manner for situations whereby interested participants, who were illiterate or with lower educational levels, requested assistance in completing the survey. Each uncontactable household was approached up to three times on different days and times of the week before being replaced with a neighbouring household of the same housing type.

The survey instrument was developed through the adaptation of other cross-sectional surveys reported in the literature, including the WHO Antibiotic Resistance: Multi-Country Public Awareness Survey [16,20,21,22,23], and was made available in the four main official languages: English, Malay, Mandarin, and Tamil. While the survey instrument included a wide range of questions such as self-reported antibiotic practices and experiences, attitudes towards antibiotics, antibiotic use, and AMR, only the question items on knowledge of antibiotic use, HIO, online HISB, eHealth literacy, healthy lifestyle behaviours, and adherence to infection prevention measures were used in this study. Basic demographic information was also included alongside.

Questions on HIO were based on a validated eight-item scale [16] presented on a 5-point Likert scale (1-Strongly disagree; 2-Disagree; 3-Neither agree nor disagree; 4-Agree; 5-Strongly agree). Responses to these eight statements were computed to a total composite score of 40, and respondents scoring 75% and above (i.e., score of 30 and above) were defined as having a high level of HIO (HL-HIO), while those with scores below 30 were defined as having a low level of HIO (LL-HIO). Knowledge questions on antibiotic use [20] were designed in the form of Yes/No or True/False/Don’t know, whereas questions on utilisation of online and social media platforms to determine online HISB and questions on eHealth literacy [22,23] were presented in the Yes/No format.

Questions on healthy lifestyle behaviours and adherence to infection prevention measures [16,21] were presented on a 5-point Likert scale (1-Never; 2-Rarely; 3-Occasionally; 4-Often; 5-Always). The questions on adherence to infection prevention measures were aligned with the Singapore Health Promotion Board’s F.I.G.H.T. campaign, which was rolled out in 2017 [24] to promote the adoption of good hygiene practices to help prevent the spread of infectious diseases through five key messages: Frequent hand washing, Immunisation, Go to the doctor, Home rest, Tissues and masks. Respondents were defined as having a high level of adoption of a healthy lifestyle when they provided all of the following responses to the five statements: “I consume fruits and vegetables” (Often/Always), “I smoke cigarettes” (Never), “I exercise at least three times a week” (Often/Always), “I take sugary drinks” (Never/Rarely/Occasionally), and “I have at least 7 hours of sleep every night” (Often/Always). Responses to infection prevention measures were combined with other infection prevention health-related questions in the questionnaire. Respondents were defined as having a high level of adherence to infection prevention measures according to national recommendations when they provided five out of six of the following responses: “Before the COVID-19 pandemic, I wash my hands with soap and water to prevent infections” (Often/Always), “I have received a flu vaccination in the last 12 months” (Yes), “Before the COVID-19 pandemic, when I feel unwell, I see a doctor to manage my symptoms” (Yes), “Before the COVID-19 pandemic, I stay at home and rest, when I have a cold/flu” (Often/Always), “I drink plenty of water, when I have a cold/flu” (Often/Always), and “Before the COVID-19 pandemic, I wear a mask when I have a cough or cold” (Often/Always).

### 2.3. Dependent Variable—Poor Knowledge of Antibiotic Use

Poor knowledge of antibiotic use was defined as not providing any of the following responses to the three statements adapted from the WHO Antibiotic Resistance: Multi-Country Public Awareness Survey [20]: “It is okay to use antibiotics that were given to a friend or family member, as long as they were used to treat the same illness” (False), “It is okay to buy the same antibiotics or request for them from a doctor, if they had helped you get better previously when you had the same symptoms” (False), and “You should stop antibiotics when you have taken all the antibiotics as directed once you have begun treatment” (Yes).

### 2.4. Data Analysis

Proportions were tabulated for categorical variables, whereas means (standard deviations, SD) were calculated for continuous variables and responses on a 5-point Likert scale. A chi-squared test was used to compare differences between proportions. A multivariable logistic regression was performed to assess for the independent association between HIO and poor knowledge of antibiotic use. Known confounders and other covariates, as selected through likelihood ratio tests and comparisons between the Akaike’s Information Criteria and Bayesian Information Criteria values, were included in the final regression model to adjust for potential confounding (Supplementary Table S1). Interactions between covariates were individually explored, and product terms were also included in the final model. Effect measure modification due to socio-demographic factors was further assessed. Statistical significance was defined as a *p*-value < 0.05. Statistical analyses were conducted in Stata version 14.0 (StataCorp LLC, College Station, TX, USA).

## 3. Results

### 3.1. Demographics of Survey Respondents

Out of 4791 households approached, 2004 (41.8%) responses were collected. Survey participants were largely similar in profile to the national population based on the 2020 Singapore Census, and the detailed breakdown is presented in Table 1.

### 3.2. Health Information Orientation

**Figure 1:**
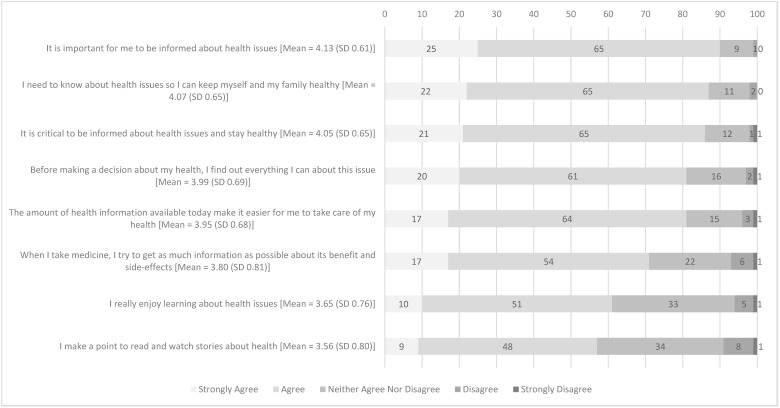
Proportion of responses (%) from 2004 respondents on statements assessing health information orientation.

The reliability of the HIO scale was high (Cronbach’s alpha = 0.8699). More than half of the respondents agreed or strongly agreed to each of the eight statements on HIO (Figure 1). A majority of the respondents agreed or strongly agreed that “it is important for me to be informed about health issues” (90%; mean = 4.13 [SD 0.61]), “I need to know about health issues so I can keep myself and my family healthy” (87%; mean = 4.07 [SD 0.65]) and “it is critical to be informed about health issues and stay healthy” (86%; mean = 4.05 [SD 0.65]). In total, 1203 (60%) respondents had HL-HIO.

### 3.3. Characteristics of Respondents with High Level of HIO vs. Low Level of HIO

Respondents with HL-HIO and LL-HIO were found to have significant differences across several characteristics (Table 2). Compared to those with LL-HIO, respondents with HL-HIO were found to be older in age (HL-HIO 21–34 years: 28%; 35–49 years: 33%; ≥50 years: 39% vs. LL-HIO: 21–34 years: 35%; 35–49 years: 32%; ≥50 years: 33%, *p* = 0.001). There was a slightly smaller representation of Chinese in the HL-HIO group (70% vs. 75%, *p* = 0.011), whilst more were currently married in the HL-HIO group (66% vs. 58%, *p* < 0.001). A larger proportion of respondents in the HL-HIO group had family members or friends working in the healthcare sector (58% vs. 47%, *p* < 0.001) and have continuity of care with a regular doctor (65% vs. 56%, *p* < 0.001). Furthermore, respondents in the HL-HIO group were more likely to self-report the influence of religion on their health-seeking behaviours (30% vs. 22%, *p* < 0.001) and self-report their health rating as above average (69% vs. 58%, *p* < 0.001). They were also more likely to display high levels of adoption of a healthy lifestyle (20% vs. 11%, *p* < 0.001) and infection prevention measures (21% vs. 13%, *p* < 0.001), as compared to those in the LL-HIO group.

### 3.4. Factors Associated with Poor Knowledge of Antibiotic Use

HIO, age, race, highest educational level, and marital status were independent factors associated with poor knowledge of antibiotic use (Table 3). Age was found to interact negatively with HIO (aged 35–49 years: aOR 0.67, 95% CI [0.42–1.06], n.s.; aged 21–34 years: aOR 0.61, 95% CI [0.39–0.97], *p* = 0.035, with reference to aged ≥50 years). Positive interactions between gender and adoption of a healthy lifestyle (Male: aOR 1.62, 95% CI [0.97–2.70], n.s.), and between self-reported influence of religion on health-seeking behaviour and continuity of care with a regular doctor (aOR 1.61, 95% CI [1.04–2.51], *p* = 0.034) were also observed. All interaction terms were included in the final model (Model 2).

After adjusting for potential confounders, respondents with LL-HIO (aOR 1.82, 95% CI [1.32–2.51], *p* < 0.001) were found to be 82% more likely to have poor knowledge of antibiotic use, as compared to those with HL-HIO. While there was an inverse dose-response relationship between age and knowledge of antibiotic use, only respondents aged 21–34 years (aOR 1.80, 95% CI [1.29–2.52], *p* = 0.001) were significantly more likely to have poor knowledge of antibiotic use, as compared to those aged ≥50 years. Non-Chinese (aOR 1.76, 95% CI [1.42–2.19], *p* < 0.001), less educated (aOR 1.86, 95% CI [1.50–2.31], *p* < 0.001), and those currently not married (aOR 1.28, 95% CI [1.04–1.57], *p* = 0.019) were also more likely to have poor knowledge of antibiotic use. Lastly, respondents without continuity of care with a regular doctor (aOR 1.17, 95% CI [0.93–1.46], n.s.) were more likely to have poor knowledge of antibiotic use.

Age modifies the effect of LL-HIO on knowledge of antibiotic use (Table 4). After adjusting for gender, race, highest educational level, marital status, continuity of care with a regular doctor, adherence to infection prevention measures, adoption of a healthy lifestyle, and self-reported influence of religion on health-seeking behaviour, we found that older adults aged ≥50 years with LL-HIO (aOR 1.81, 95% CI [1.32–2.51], *p* < 0.001) were 80% more likely to have poor knowledge of antibiotic use than those with HL-HIO in the same age group. Furthermore, we found that among individuals who self-reported influence of religion on their health-seeking behaviour, those who lacked continuity of care with a regular doctor (aOR 1.89, 95% CI [1.28–2.77], *p* = 0.001) were almost twice as likely to have poor knowledge of antibiotic use as those with continuity of care with a regular doctor (Table 5).

### 3.5. Online Health Information-Seeking Behaviours (HISBs) among Respondents with High and Low Levels of HIO

Respondents with HL-HIO were more likely to use the Internet for health-related information, as compared to those with a LL-HIO (Supplementary Table S2). Furthermore, respondents with HL-HIO also showed better self-efficacy, in particular, the ability to differentiate high-quality health resources from low-quality health resources on the Internet (60% vs. 44%, *p* < 0.001), and having the confidence to use information from the Internet to make health decisions (53% vs. 37%, *p* < 0.001).

**Figure 2:**
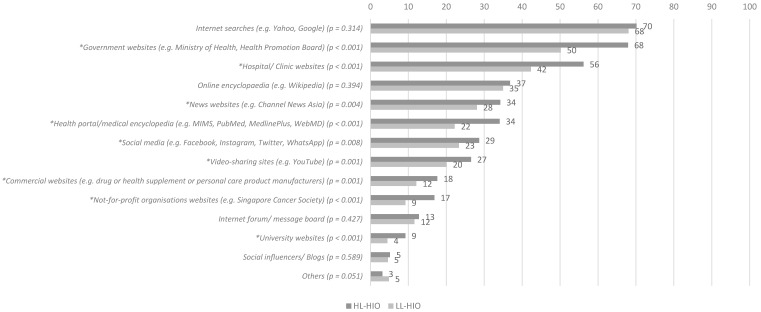
Proportion (%) of 2004 respondents who utilised the following online platforms to seek health information. * indicates a statistically significant (*p* < 0.05) difference in proportions of respondents utilising each online platform to seek health information between HL-HIO and LL-HIO groups.

Among those who sought health information online, the top three sources were Internet searches (69%), followed by government websites (61%), and hospitals or clinic websites (51%). Compared to respondents with LL-HIO, a significantly larger proportion of respondents with HL-HIO utilised online platforms including government websites (68% vs. 50%, *p* < 0.001) and hospital or clinic websites (56% vs. 42%, *p* < 0.001) (Figure 2).

When stratified by age, respondents with LL-HIO and aged ≥50 years were least likely to utilise Internet searches (53% vs. 77% vs. 75%, *p* < 0.001) and hospital or clinic websites (33% vs. 46% vs. 47%, *p* = 0.001), as compared to respondents with LL-HIO but aged 35–49 years and 21–34 years, respectively (Supplementary Table S4). Of interest, there were no significant differences between age groups among respondents with LL-HIO on their utilisation of government websites to seek health information online (21–34 years old: 52%; 35–49 years old: 54%; ≥50 years old: 45%, *p* = 0.084).

## 4. Discussion

Our study has provided valuable insights into the positive association between HIO and knowledge of antibiotic use. Compared to individuals with HL-HIO, individuals with LL-HIO were 82% more likely to have a poor knowledge of antibiotic use. Previous studies have shown the effects of HIO on the adoption of behaviours, such as prevention against infection with the Middle East Respiratory Syndrome and on the use of health applications [25,26], but the association between HIO and health knowledge is novel. We have further elucidated the profiles of individuals with LL-HIO for targeted educational interventions. They tended to be younger in age, not currently married, without family members or friends working in the healthcare sector, and without continuity of care with a regular doctor. Younger adults tend to have diverse relationships and wide-ranging sources of information and are less likely to have healthcare encounters [27,28,29,30]. Although healthcare providers are their most trusted sources of health information, there is a need to identify and harness non-healthcare related influencers within their vast network of social contacts who could serve as trusted sources of health information [31,32,33].

Other characteristics more likely to be associated with LL-HIO are self-reported non-influence of religion on health-seeking behaviour, average or below average self-reported health rating, low-level of adoption of a healthy lifestyle and low adherence to infection prevention measures. The findings are concerning and there are calls for action to reach out to individuals with LL-HIO via passive channels of communication frequented by them, not only to increase their knowledge on antibiotic use and consequently antibiotic practice, but also to increase their adoption of healthy lifestyle and infection prevention behaviours. A previous study has shown that individuals with LL-HIO preferred passive information sources (such as the television and radio) to the Internet or newspapers/magazines [20]. Furthermore, based on the findings from a 10-year population-based survey conducted in Sweden, lower socio-economic status and lack of social support and informal caregiving were associated with poor health-seeking behaviour, i.e., seeking medical care in the study context [34]. Hence, there is a need for deeper and more extensive social outreach to actively disseminate health information, for example on appropriate antibiotic use and AMR, to these sub-populations, as they are less likely to present themselves to a healthcare facility or have interest to actively seek out health information. Contemporary communication channels such as social media posts by trusted healthcare providers, with interactive games and videos embedded could be considered to provide health information to this sub-population with LL-HIO [35].

Having LL-HIO was found to have a strong association with poor knowledge of antibiotic use, especially in adults aged ≥50 years. Only around 50% of this specific sub-population was observed to be utilising the Internet to obtain information on diseases, medications, illness and health management (Supplementary Table S3). As such, a low proportion of them reported the use of Internet-based platforms to look for health information (Supplementary Table S4), which could be attributable to poor eHealth or health literacy [15]. More than two-thirds of them were unable to differentiate the quality of Internet health resources and lacked the confidence to use these Internet-derived information to make health decisions. This suggests that for the purposes of disseminating health information, Internet-based platforms are less effective for older adults with LL-HIO due to the much lower Internet utilisation rates and barriers to utilise Internet-based information. Instead, there is a need for physical, social and healthcare touchpoints, with whom they have close and sustained relationships, to actively reach out to and educate this sub-population, using traditional non-Internet-based modalities such as posters or pamphlets and annual campaigns on appropriate antibiotic use to improve antibiotic practices [14]. Similar findings on the relationship between HIO, health information efficacy, and community participation in good preventive health behaviours have been previously reported, highlighting the importance of working with non-traditional communication platforms to reach out to specific segments of the community [36].

An interesting observation was that the continuity of care with a regular doctor halves (47%) the odds of having poor knowledge of antibiotic use in respondents who self-reported religious influence on their health-seeking behaviour, as compared to counterparts without continuity of care with a regular doctor. A study conducted in the Netherlands reported that individuals with a cultural predisposition to mistrust in information sources and have low concerns on AMR gained more awareness from an educational video developed by the National Institute for Public Health and the Environment than those who were culturally predisposed to trust and have high concerns on AMR [37]. This highlights the critical role of health authorities and healthcare professionals, such as doctors, as trusted sources of health information, and the need to empower them with tools to provide patients with accurate information on antibiotic use and AMR, through building trusting doctor-patient relationships established through continuity of care [38].

Furthermore, adults aged 21–34 years were also found to be more likely to have poor knowledge of antibiotic use. Such poor knowledge in these younger adults was previously shown to be associated with increased odds of having poor antibiotic practices by a factor of 6.6 [14]. While young adults were more likely to utilise Internet searches, government websites and hospital or clinic websites to seek health information, were confident in using the Internet to answer questions about health, and able to differentiate between high and low quality online health information (*p* < 0.05), their low-levels of HIO would hinder them from actively seeking out health information. Therefore, it is important for government bodies (e.g., the Ministry of Health and the Health Promotion Board), hospitals and clinics to utilise one-stop online platforms to periodically push out accurate, consistent and comprehensive health information, including that on appropriate antibiotic use [15] to avoid the need for these younger adults to have to take the extra effort to seek out information on antibiotic use.

Overall, our study supports existing literature that “one-size-fits-all” nationwide AMR campaigns are unlikely to be effective in raising awareness and improving antibiotic knowledge and practices [12,13], and highlights the importance of employing the right communication strategies for specific subpopulations due to the diversity in HIO profiles. Future AMR interventions should adopt a multi-pronged approach and employ various online and offline communications platforms and modalities, harnessing traditional and non-traditional healthcare and non-healthcare influencers.

This study has several strengths. Firstly, the proportionately stratified random sampling methodology avoided potential selection bias and cluster bias, and together with the oversampling of minority groups, a nationally representative sample population was achieved. The adaptation and incorporation of questions from validated scales and well-established questionnaires such as the eHEALS: eHealth literacy scale and WHO’s Antibiotic Resistance: Multi-Country Public Awareness Survey added to the reliability of the findings and allowed for international comparisons. The provision of survey booklets in the four main official languages in Singapore and the option for interviewer administration of the surveys further minimised selection bias by literacy and educational levels. Surveyors were also trained prior to data collection to prevent interviewer bias. Furthermore, this is the first nationally-representative survey conducted to profile HIO and assess the association of HIO with knowledge of antibiotic use to guide future interventions. However, we cannot exclude under- or over-reporting of responses due to social desirability bias, although it is likely to be reduced due to the anonymity of the survey. Also, there might be unknown and unmeasured confounders not included in the final multivariable logistic regression model. Although we are unable to draw conclusions on causality due to the cross-sectional nature of the study, we have found a strong association between HIO and knowledge of antibiotic use.

## 5. Conclusions

Poor knowledge on antibiotic use is associated with poor antibiotic use practices. Using a nationally representative population, we demonstrated that LL-HIO is independently associated with poor knowledge of antibiotic use, and further elucidated the profiles of individuals with LL-HIO and their online HISB. Older adults (≥50 years) with LL-HIO were most likely to have poor knowledge on antibiotic use and would benefit most from education via non-Internet-based passive information sources including regular healthcare providers and influencers within their social networks.

## Figures and Tables

**Table 1:** Characteristics of 2004 Singapore residents surveyed between November 2020 and January 2021.

Characteristics	Survey Respondents, %	Singapore Residents in Census 2020 ^a^, %
***Residency Status, N(%)***		
Singapore Citizen	87	86
Permanent Resident	13	14
***Age, N(%)***		
21–34 years old	31	26
35–49 years old	33	28
≥50 years old	36	46
***Gender, N(%)***		
Male	48	48
Female	52	52
***Race, N(%)***		
Chinese	72	76
Non-Chinese	28	24
***Highest Educational Level, N(%)***		
Lower Educated (Post-Secondary and Below)	35	51
Higher Educated (Diploma and Above)	65	49
***Marital Status, N(%)***		
Currently Married	62	63
Currently Not Married	38	37
***Employment Status, N(%)***		
Currently Employed	70	NA
Currently Not Employed	30	NA
***Self-Reported Influence of Religion on Health-Seeking Behaviour, N(%)***		
Yes	27	NA
***Have Family Members or Friends Working in Healthcare Sector, N(%)***		
Yes	54	NA
***Have At Least One Chronic Disease, N(%)***		
Yes	32	NA
***Self-Reported Health Rating, N(%)***		
Below Average	3	NA
Average	33	NA
Above Average	65	NA
***Adoption of Healthy Lifestyle, N(%)***		
High	16	NA
Low	84	NA
***Continuity of Care with a Regular Doctor, N(%)***		
Yes	62	NA
***Adherence to Infection Prevention Measures, N(%)***		
High	18	NA
Low	83	NA

^a^ Includes population who are 20 years and above.

**Table 2:** Characteristics of 2004 respondents with high level of HIO vs. low level of HIO.

Characteristics	High Level of Health Information Orientation (N = 1203)	Low Level of Health Information Orientation (N = 801)	*p*-Value *
***Residency Status, N(%)***			
Singapore Citizen	1048 (87)	690 (86)	0.529
Permanent Resident	155 (13)	111 (14)	
***Age, N(%)***			
21–34 years old	334 (28)	281 (35)	**0.001**
35–49 years old	402 (33)	256 (32)	
≥50 years old	467 (39)	264 (33)	
***Gender, N(%)***			
Male	558 (46)	396 (49)	0.180
Female	645 (54)	405 (51)	
***Race, N(%)***			
Chinese	838 (70)	600 (75)	**0.011**
Non-Chinese	365 (30)	201 (25)	
***Highest Educational Level, N(%)***			
Lower Educated (Post-Secondary and Below)	418 (35)	278 (35)	0.985
Higher Educated (Diploma and Above)	785 (65)	523 (65)	
***Marital Status, N(%)***			
Currently Married	790 (66)	462 (58)	**<0.001**
Currently Not Married	413 (34)	339 (42)	
***Employment Status, N(%)***			
Currently Employed	372 (31)	228 (28)	0.239
Currently Not Employed	831 (69)	573 (72)	
***Self-Reported Influence of Religion on Health-Seeking Behaviour, N(%)***			
Yes	363 (30)	176 (22)	**<0.001**
***Have Family Members or Friends Working in Healthcare Sector, N(%)***			
Yes	702 (58)	374 (47)	**<0.001**
***Have At Least One Chronic Disease, N(%)***			
Yes	404 (34)	244 (30)	0.143
***Self-Reported Health Rating, N(%)***			
Below Average	30 (2)	20 (3)	**<0.001**
Average	342 (28)	319 (40)	
Above Average	831 (69)	462 (58)	
***Adoption of Healthy Lifestyle, N(%)***			
High	245 (20)	85 (11)	**<0.001**
Low	958 (80)	716 (89)	
***Continuity of Care with a Regular Doctor, N(%)***			
Yes	784 (65)	449 (56)	**<0.001**
***Adherence to Infection Prevention Measures, N(%)***			
High	247 (21)	106 (13)	**<0.001**
Low	956 (79)	965 (87)	

* Bolded values indicate statistical significance of *p* < 0.05.

**Table 3:** Univariate and multivariable logistic regression analyses on factors associated with knowledge of antibiotic use (N = 2004).

Variables	Good Knowledge of Antibiotic Use (N = 1188)	Poor Knowledge of Antibiotic Use (N = 816)	*p*-Value *	Univariate Analysis (N = 2004)		Model 1: without Interaction Terms (N = 2004)		Model 2: with Interaction Terms (N = 2004)	
				Odds Ratio (95% CI)	*p*-Value *	Adjusted Odds Ratio (95% CI)	*p*-Value *	Adjusted Odds Ratio (95% CI)	*p*-Value *
***Level of Health Information Orientation, N(%)***									
Low	438 (37)	363 (44)	**0.001**	1.37 (1.14–1.65)	**0.001**	1.36 (1.12–1.65)	**0.002**	1.82 (1.32–2.51)	**<0.001**
***High Adherence to Infection Prevention Measures, N(%)***									
Yes	191 (16)	162 (20)	**0.029**	1.29 (1.03–1.63)	**0.030**	1.22 (0.96–1.56)	0.109	1.21 (0.95–1.55)	0.123
***Residency Status, N(%)***									
Permanent Resident	172 (14)	94 (12)	0.055	Ref	-	-	-	-	-
Singapore Citizen	1016 (86)	722 (88)		1.30 (0.99–1.70)	0.056	-	-	-	-
***Age, N(%)***									
≥50 years old	446 (38)	285 (35)	**0.001**	Ref	-	Ref	-	Ref	-
35–49 years old	416 (35)	242 (30)		0.91 (0.73–1.13)	0.397	1.08 (0.85–1.37)	0.535	1.27 (0.94–1.72)	0.124
21–34 years old	326 (27)	289 (35)		1.39 (1.12–1.72)	**0.003**	1.47 (1.12–1.92)	**0.006**	1.80 (1.29–2.52)	**0.001**
***Gender, N(%)***									
Male	529 (45)	425 (52)	**0.001**	1.35 (1.13–1.62)	**0.001**	1.36 (1.13–1.64)	**0.001**	0.90 (0.56–1.44)	0.651
***Race, N(%)***									
Non-Chinese	267 (22)	299 (37)	**<0.001**	1.99 (1.64–2.43)	**<0.001**	1.77 (1.43–2.20)	**<0.001**	1.76 (1.42–2.19)	**<0.001**
***Highest Educational Level, N(%)***									
Higher Educated (Diploma and Above)	838 (71)	470 (58)	**<0.001**	Ref	-	Ref	-	Ref	-
Lower Educated (Post-Secondary and Below)	350 (29)	346 (42)		1.76 (1.46–2.12)	**<0.001**	1.90 (1.53–2.36)	**<0.001**	1.86 (1.50–2.31)	**<0.001**
***Employment Status, N(%)***									
Currently Not Employed	344 (29)	256 (31)	0.246	1.12 (0.92–1.36)	0.246	-	-	-	-
***Marital Status, N(%)***									
Currently Not Married	409 (34)	343 (42)	**0.001**	1.38 (1.15–1.66)	**0.001**	1.28 (1.05–1.58)	**0.017**	1.28 (1.04–1.57)	**0.019**
***Self-Reported Influence of Religion on Health-Seeking Behaviour, N(%)***									
Yes	293 (25)	246 (30)	**0.007**	1.32 (1.08–1.61)	**0.007**	1.20 (0.97–1.49)	0.100	1.03 (0.79–1.35)	0.814
***Have Family Members or Friends Working in Healthcare Sector, N(%)***									
No	531 (45)	397 (49)	0.081	1.17 (0.98–1.40)	0.081	-	-	-	-
***Have At Least One Chronic Disease, N(%)***									
Yes	381 (32)	267 (33)	0.760	1.03 (0.85–1.25)	0.760	-	-	-	-
***Self-Reported Health Rating, N(%)***									
Below Average	36 (3)	14 (2)	0.170	Ref	-	-	-	-	-
Average	393 (33)	268 (33)		1.75 (0.93–3.31)	0.084	-	-	-	-
Above Average	759 (64)	534 (65)		1.81 (0.97–3.39)	0.064	-	-	-	-
***Adoption of Healthy Lifestyle, N(%)***									
Low	973 (82)	701 (86)	**0.018**	1.35 (1.05–1.72)	**0.018**	1.09 (0.84–1.41)	0.516	0.90 (0.56–1.44)	0.414
***Continuity of Care with a Regular Doctor, N(%)***									
No	418 (35)	353 (43)	**<0.001**	1.40 (1.17–1.69)	**<0.001**	1.32 (1.09–1.61)	**0.005**	1.17 (0.93–1.46)	0.171
***Interaction between Health Information Orientation and Age***									
Low health information orientation and 35–49 years old	-	-	-	0.63 (0.40–0.99)	**0.044**	-	-	0.67 (0.42–1.06)	0.084
Low health information orientation and 21–34 years old	-	-	-	0.58 (0.37–0.91)	**0.016**	-	-	0.61 (0.39–0.97)	**0.035**
***Interaction between Adoption of Healthy Lifestyle and Gender***									
Low adoption of healthy lifestyle and male gender	-	-	-	1.72 (1.05–2.82)	**0.033**	-	-	1.62 (0.97–2.70)	0.067
***Interaction between Continuity of Care with a Regular Doctor and Self-Reported Influence of Religion on Health-Seeking Behaviour***									
Lack of continuity of care with a regular doctor and self-reported influence of religion on health-seeking behaviour	-	-	-	1.87 (1.22–2.86)	**0.004**	-	-	1.61 (1.04–2.51)	**0.034**

* Bolded values indicate statistical significance of *p* < 0.05.

**Table 4:** Association between poor knowledge of antibiotic use and health information orientation, according to age group (N = 2004).

Poor Knowledge of Antibiotic Use	≥50 Years Old(N = 731)			35–49 Years Old(N = 658)			21–34 Years Old(N = 615)		
	OR	95% CI	*p*-Interaction ^a,^*	OR	95% CI	*p*-Interaction ^a^	OR	95% CI	*p*-Interaction ^a^
**Unadjusted analysis**									
High health information orientation	Ref	-	**<0.001**	Ref	-	0.332	Ref	-	0.632
Low health information orientation	1.86	1.37–2.53		1.17	0.85–1.62		1.08	0.79–1.49	
**Adjusted analysis ^b^**									
High health information orientation	Ref	-	**<0.001**	Ref	-	0.265	Ref	-	0.534
Low health information orientation	1.81	1.32–2.51		1.21	0.87–1.69		1.11	0.80–1.55	

^a^ Multiplicative scale; ^b^ Adjusted for gender, race, highest educational level, marital status, continuity of care with a regular doctor, adherence to infection prevention measures, adoption of healthy lifestyle, and self-reported influence of religion on health-seeking behaviour. * Bolded values indicate statistical significance of *p* < 0.05.

**Table 5:** Association between poor knowledge of antibiotic use and continuity of care with a regular doctor, according to self-reported influence of religion on health-seeking behaviour (N = 2004).

Poor Knowledge of Antibiotic Use	Lack of Self-Reported Influence of Religion on Health-Seeking Behaviour(N = 539)			Presence of Self-Reported Influence of Religion on Health-Seeking Behaviour(N = 1465)		
	OR	95% CI	*p*-Interaction ^a^	OR	95% CI	*p*-Interaction ^a,^*
**Unadjusted analysis**						
With continuity of care with a regular doctor	Ref	-	0.065	Ref	-	**<0.001**
Without continuity of care with a regular doctor	1.22	0.99–1.51		2.29	1.58–3.30	
**Adjusted analysis ^b^**						
With continuity of care with a regular doctor	Ref	-	0.171	Ref	-	**0.001**
Without continuity of care with a regular doctor	1.17	0.93–1.46		1.89	1.28–2.77	

^a^ Multiplicative scale; ^b^ Adjusted for age, gender, race, highest educational level, marital status, health information orientation, adherence to infection prevention measures, and adoption of healthy lifestyle. * Bolded values indicate statistical significance of *p* < 0.05.

## Data Availability

The datasets used and/or analysed during the current study are available from the corresponding author on reasonable request.

## Supplementary Materials

The following supporting information can be downloaded at: https://www.mdpi.com/article/10.3390/antibiotics11060769/s1, Table S1: Model and variable selection for final multivariable logistic regression model assessing factors associated with poor knowledge of antibiotic use; Table S2: Proportion of 2004 respondents who agreed to the following statements on the use of the Internet to search for information on health-related matters, stratified by health information orientation; Table S3: Proportion of 2004 respondents who agreed to the following statements on the use of the Internet to search for information on health-related matters, stratified by health information orientation and age group; Table S4: Proportion of 2004 Singapore residents using different Internet-based platforms to look for health information, stratified by health information orientation and age group.
