# Supplementary material for: Health Information Orientation Profiles and Their Association with Knowledge of Antibiotic Use in a Population with Good Internet Access: A Cross-Sectional Study
Source: Antibiotics (Basel). 2022 Jun 4;11(6):769. doi: 10.3390/antibiotics11060769 (PMC9220153; doi:10.3390/antibiotics11060769)
Supplement: Supplementary file 1 [file antibiotics-11-00769-s001.zip › antibiotics-1754469-SI.pdf]

Supplementary Table S1: Model and variable selection for final multivariable logistic regression model assessing factors associated with poor knowledge of antibiotic use.

Variables in the model		AIC	BIC	Likelihood ratio	*p*-value
M1	Demographics (Age, Gender, Race, Highest Educational Level, Marital Status)	2604.989	2644.210	Ref	-
M2	Demographics + Health Information Orientation	2596.526	2641.349	10.46	0.001
M3	Demographics + Health Information Orientation + Health Information Orientation*Age interaction	2594.835	2650.864	16.15	0.001
M4	Demographics + Health Information Orientation + Health Information Orientation*Age interaction + Continuity of Care with a Regular Doctor	2590.607	2652.239	22.38	<0.001
M5	Demographics + Health Information Orientation + Health Information Orientation*Age interaction + Continuity of Care with a Regular Doctor + Adherence to At Least 5 Infection Prevention Measures	2590.036	2657.271	24.95	<0.001
M6	Demographics + Health Information Orientation + Health Information Orientation*Age interaction + Continuity of Care with a Regular Doctor + Adherence to At Least 5 Infection Prevention Measures + Adoption of Healthy Lifestyle	2591.721	2664.559	25.27	<0.001
M7	Demographics + Health Information Orientation + Health Information Orientation*Age interaction + Continuity of Care with a Regular Doctor + Adherence to At Least 5 Infection Prevention Measures + Adoption of	2590.285	2668.726	28.70	<0.001

	Healthy Lifestyle + Adoption of Healthy Lifestyle*Gender interaction				
M8	Demographics + Health Information Orientation + Health Information Orientation*Age interaction + Continuity of Care with a Regular Doctor + Adherence to At Least 5 Infection Prevention Measures + Adoption of Healthy Lifestyle + Adoption of Healthy Lifestyle*Gender interaction + Self-Reported Influence of Religion on Health-Seeking Behaviour	2589.168	2673.211	31.82	<0.001
**M9**	**Demographics + Health Information Orientation + Health Information Orientation*Age interaction + Continuity of Care with a Regular Doctor + Adherence to At Least 5 Infection Prevention Measures + Adoption of Healthy Lifestyle + Adoption of Healthy Lifestyle*Gender interaction + Self-Reported Influence of Religion on Health-Seeking Behaviour + Continuity of Care with a Regular Doctor*Self-Reported Influence of Religion on Health-Seeking Behaviour interaction**	**2586.664**	**2676.31**	**36.33**	**<0.001**

** The final selected model is highlighted in bold.*

Supplementary Table S2: Proportion of 2004 respondents who agreed to the following statements on the use of the Internet to search for information on health-related matters, stratified by health information orientation.

**Statements**	**High Level of Health Information Orientation (N = 1203)**	**Low Level of Health Information Orientation (N = 801)**	***p*-value***
I use the Internet to get description of various diseases	83	75	**<0.001**
I use the Internet to get information on medicine/drugs	80	69	**<0.001**
I use the Internet to understand how to deal with an illness	80	70	**<0.001**
I use the Internet to get information on health management	86	73	**<0.001**
I know how to use the Internet to answer my questions about health	79	68	**<0.001**
I can tell high-quality health resources from low-quality health resources on the Internet	60	44	**<0.001**
I feel confident in using information from the Internet to make health decisions	53	37	**<0.001**

** Bolded values indicate statistical significance of $p < 0.05$.*

Supplementary Table S3: Proportion of 2004 respondents who agreed to the following statements on the use of the Internet to search for information on health-related matters, stratified by health information orientation and age group.

**Statements, N(%)**	**Total (N = 2004)**	**High Level of Health Information Orientation (N = 1203)**				**Low Level of Health Information Orientation (N = 801)**			
		**21-34 years old (N = 334)**	**35-49 years old (N = 402)**	**≥50 years old (N = 467)**	***p*-value***	**21-34 years old (N = 281)**	**35-49 years old (N = 256)**	**≥50 years old (N = 264)**	***p*-value***
I use the Internet to get description of various diseases	1600 (80)	302 (90)	356 (89)	339 (73)	**<0.001**	226 (80)	256 (86)	157 (59)	**<0.001**
I use the Internet to get information on medicine/drugs	1519 (76)	297 (89)	347 (86)	323 (69)	**<0.001**	222 (79)	193 (75)	137 (52)	**<0.001**
I use the Internet to understand how to deal with an illness	1521 (76)	304 (91)	335 (83)	320 (69)	**<0.001**	221 (79)	206 (80)	135 (51)	**<0.001**
I use the Internet to get information on health management	1618 (81)	319 (96)	371 (92)	342 (73)	**<0.001**	227 (81)	206 (80)	153 (58)	**<0.001**
I know how to use the Internet to answer my questions about health	1493 (75)	297 (89)	331 (82)	319 (68)	**<0.001**	218 (78)	194 (76)	134 (51)	**<0.001**
I can tell high-quality health resources from low-quality health resources on the Internet	1077 (54)	241 (72)	249 (62)	232 (50)	**<0.001**	146 (52)	125 (49)	84 (32)	**<0.001**
I feel confident in using information from the Internet to make health decisions	936 (47)	195 (58)	216 (54)	230 (49)	**0.037**	114 (41)	106 (41)	75 (28)	**0.002**

** Bolded values indicate statistical significance of p < 0.05.*

Supplementary Table S4: Proportion of 2004 Singapore residents using different Internet-based platforms to look for health information, stratified by health information orientation and age group.

Platforms, N(%)	Total (N = 2004)	High Level of Health Information Orientation (N = 1203)				Low Level of Health Information Orientation (N = 801)			
		**21-34 years old (N = 334)**	**35-49 years old (N = 402)**	**≥50 years old (N = 467)**	***p*-value***	**21-34 years old (N = 281)**	**35-49 years old (N = 256)**	**≥50 years old (N = 264)**	***p*-value***
Internet searches (e.g. Yahoo, Google)	1389 (69)	252 (75)	306 (76)	286 (61)	**<0.001**	210 (75)	196 (77)	139 (53)	**<0.001**
Government websites (e.g. Ministry of Health, Health Promotion Board)	1219 (61)	236 (71)	292 (73)	289 (62)	**0.001**	146 (52)	138 (54)	118 (45)	0.084
Hospital / Clinic websites	1015 (51)	217 (65)	227 (56)	232 (50)	**<0.001**	132 (47)	119 (46)	88 (33)	**0.001**
Online encyclopedia (e.g. Wikipedia)	723 (36)	137 (41)	168 (42)	138 (30)	**<0.001**	108 (38)	107 (42)	65 (25)	**<0.001**
News websites (e.g. Channel News Asia)	637 (32)	145 (43)	140 (35)	127 (27)	**<0.001**	86 (31)	84 (33)	55 (21)	**0.005**
Health portal/medical encyclopedia (e.g. MIMS, PubMed, MedlinePlus, WebMD)	588 (29)	129 (39)	149 (37)	132 (28)	**0.003**	71 (25)	68 (27)	39 (15)	**0.002**
Social media (e.g. Facebook, Instagram, Twitter, WhatsApp)	532 (27)	112 (34)	113 (28)	120 (26)	0.051	72 (26)	67 (26)	48 (18)	0.053

Video-sharing sites (e.g. YouTube)	480 (24)	103 (31)	101 (25)	115 (25)	0.108	58 (21)	56 (22)	47 (18)	0.491
Commercial websites (e.g. drug or health supplement or personal care product manufacturers)	309 (15)	60 (18)	76 (19)	76 (16)	0.586	35 (12)	33 (13)	29 (11)	0.782
Not-for-profit organizations websites (e.g. Singapore Cancer Society)	277 (14)	55 (16)	72 (18)	76 (16)	0.792	28 (10)	27 (11)	19 (7)	0.366
Internet forum / message board	247 (12)	42 (13)	59 (15)	53 (11)	0.339	35 (12)	36 (14)	22 (8)	0.108
University websites	147 (7)	42 (13)	41 (10)	28 (6)	**0.005**	15 (5)	11 (4)	10 (4)	0.672
Social influencers / Blogs	99 (5)	27 (8)	17 (4)	18 (4)	**0.017**	18 (6)	13 (5)	6 (2)	0.065
Other non-Internet-based platforms/media	77 (4)	11 (3)	5 (1)	22 (5)	**0.014**	3 (1)	4 (2)	32 (12)	**<0.001**

** Bolded values indicate statistical significance of p < 0.05.*
